# First Insights into the Large Genome of *Epimedium sagittatum* (Sieb. et Zucc) Maxim, a Chinese Traditional Medicinal Plant

**DOI:** 10.3390/ijms140713559

**Published:** 2013-06-27

**Authors:** Di Liu, Shao-Hua Zeng, Jian-Jun Chen, Yan-Jun Zhang, Gong Xiao, Lin-Yao Zhu, Ying Wang

**Affiliations:** 1 Key Laboratory of Plant Germplasm Enhancement and Specialty Agriculture, Wuhan Botanical Garden, Chinese Academy of Sciences, Wuhan 430074, China; E-Mails: liudi206@mails.ucas.ac.cn (D.L.); jianjunchen@wbgcas.cn (J.-J.C.); yanjunzhang@wbgcas.cn (Y.-J.Z.); gongxiaobio@gmail.com (G.X.); 2 University of Chinese Academy of Sciences, Beijing 100039, China; 3 Key Laboratory of Plant Resources Conservation and Sustainable Utilization, South China Botanical Garden, Chinese Academy of Sciences, Guangzhou 510650, China; E-Mail: shhzeng@scib.ac.cn; 4 Wuhan Vegetable Research Station, Wuhan 430065, China; E-Mail: zly01062535@163.com

**Keywords:** *Epimedium sagittatum*, Chinese medicinal plant, structural genomics, genome composition, *Gypsy-Ty3* retrotransposon, repetitive elements, FISH, chromosome

## Abstract

*Epimedium sagittatum* (Sieb. et Zucc) Maxim is a member of the Berberidaceae family of basal eudicot plants, widely distributed and used as a traditional medicinal plant in China for therapeutic effects on many diseases with a long history. Recent data shows that *E. sagittatum* has a relatively large genome, with a haploid genome size of ~4496 Mbp, divided into a small number of only 12 diploid chromosomes (2*n* = 2*x* = 12). However, little is known about *Epimedium* genome structure and composition. Here we present the analysis of 691 kb of high-quality genomic sequence derived from 672 randomly selected plasmid clones of *E. sagittatum* genomic DNA, representing ~0.0154% of the genome. The sampled sequences comprised at least 78.41% repetitive DNA elements and 2.51% confirmed annotated gene sequences, with a total GC% content of 39%. Retrotransposons represented the major class of transposable element (TE) repeats identified (65.37% of all TE repeats), particularly LTR (Long Terminal Repeat) retrotransposons (52.27% of all TE repeats). Chromosome analysis and Fluorescence *in situ* Hybridization of *Gypsy-Ty3* retrotransposons were performed to survey the *E. sagittatum* genome at the cytological level. Our data provide the first insights into the composition and structure of the *E. sagittatum* genome, and will facilitate the functional genomic analysis of this valuable medicinal plant.

## 1. Introduction

*Epimedium* L. (“*Yin Yang Huo*” in Chinese) is a genus of the Berberidaceae family, endemic to and widely distributed in China [1]. Species of *Epimedium* have been used as traditional medicinal plants in China and East Asia for more than 2000 years. To date, five species have been recorded in the Chinese Pharmacopoeia as medicinal plants: *E. brevicornu* Maxim, *E. sagittatum* (Sieb. et Zucc) Maxim, *E. pubescens* Maxim, *E. wushanense* T. S. Ying and *E. koreanum* Nakai (Chinese Pharmacopoeia Commission, 2005) [2]. The significant worth of these *Epimedium* species to traditional medicine is attributed largely to their high levels of bioactive chemicals, especially flavonoids [3], which play an important role in cell senescence delay [4] and retarding aging [5]. In addition, *Epimedium* has attracted increasing commercial attention for its use in the treatment of impotence, spermatorrhea, infertility, amenorrhea and menopause symptoms [6].

To date, research into *Epimedium* species has concentrated on taxonomy and phytogeography [7,8], phylogenetic analysis [9–11], and chemical and pharmacological investigations [3–6,8]. More recently, the development of an EST dataset and EST-SSRs in *E. sagittatum* [12] combined with characterization of the genes involved in flavonoid biosynthesis [13,14] and the evolution of carpel and nectary development in this basal eudicot [15], have shed new light on *Epimedium* functional genomics. Fundamentally, however, our understanding of the basic genomic characteristics of the genus *Epimedium*, such as genome size and genome structure, is still lacking

Given that *Epimedium* is a genus of Berberidaceae, a member of Ranunculales lying in a basal clade of eudicots, research into *Epimedium* genomics has the potential to enhance our understanding of the phylogeny and evolution of angiosperms, from which almost all modern cultivated species are exclusively derived. Therefore, given not only the great medicinal value, but also the important placement in evolutionary history, of *Epimedium*, investigation of the *Epimedium* genome is both warranted and timely.

All *Epimedium* species analysed to date have only 12 chromosomes (2*n* = 2*x* = 12) [16], but genome sizes range from 4115.35 Mbp/1C to 4876.4 Mbp/1C, giving a median haploid genome size of ~4496 Mbp [17]. This genome size is relatively large compared to model plant species such as *Arabidopsis thaliana* (156 Mbp/1C) [18], *Oryza sativa* (466 Mbp/1C) [19], *Carica papaya* (372 Mbp/1C) [20], *Vitis vinifera* (475Mbp/1C) [21], *Medicago truncatula* (465 Mbp/1C) [20], *Populus trichocarpa* (484 Mbp/1C) [22], *Gossypium raimondii* (880 Mbp/1C) [23], *Solanum lycopersicon* (900 Mbp/1C) [24] and even *Zea mays* (2,665 Mbp/1C) [19]. Therefore, despite advances in genome sequencing technologies and analysis techniques, it remains comparatively expensive to sequence the whole genome of *Epimedium*. Furthermore, genome size in plants is usually positively correlated with an increasing content of repetitive DNA, mainly transposable elements (TEs) [25], making *de novo* assembly of next generation sequence reads particularly difficult for large genomes. Instead, for investigating large genomes, the GeneTrek approach, first used in analyzing the pufferfish (*Fugu*) genome, is considered feasible and cost-effective [26]. This approach works by first randomly sequencing and annotating a small subsection of the genome and then extrapolating this information to estimate the characteristics of the genome in general [27]. This has been widely adopted to provide the first insights into genomes such as maize [27–29], rye [30], cotton [31], papaya [32], bread wheat [33], chickpea [34], woodland strawberry [35], and carrot [36], as well as the medicinal gymnosperm *Taxus mairei* [37].

Here we report the first structural analysis of the *E. sagittatum* genome using the GeneTrek approach combined with chromosome analysis and FISH of *Gypsy-Ty3* retrotransposable elements. The information gained in this study lays the foundations for future research into *Epimedium* genome organization, species evolution and also the functional analysis of genes involved in the complex and valuable metabolism of *Epimedium* species.

## 2. Results

### 2.1. Sequence Assembly and Composition

**Figure 1:**
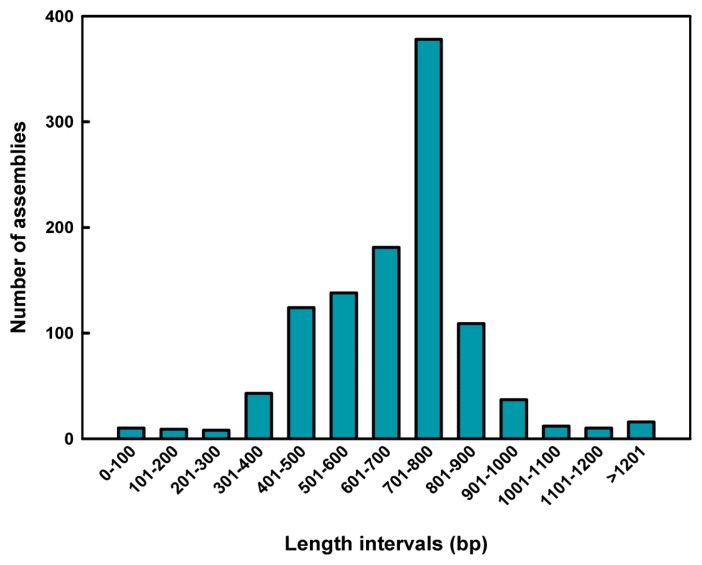
Length distribution of insert sequences of plasmid clones. Sequencing of the 672 randomly selected clones generated 1075 assemblies (126 contigs, 949 singlets). The *x*-axis indicates the length interval of insert sequences. The *y*-axis indicates the number of assemblies at each length interval.

**Figure 2:**
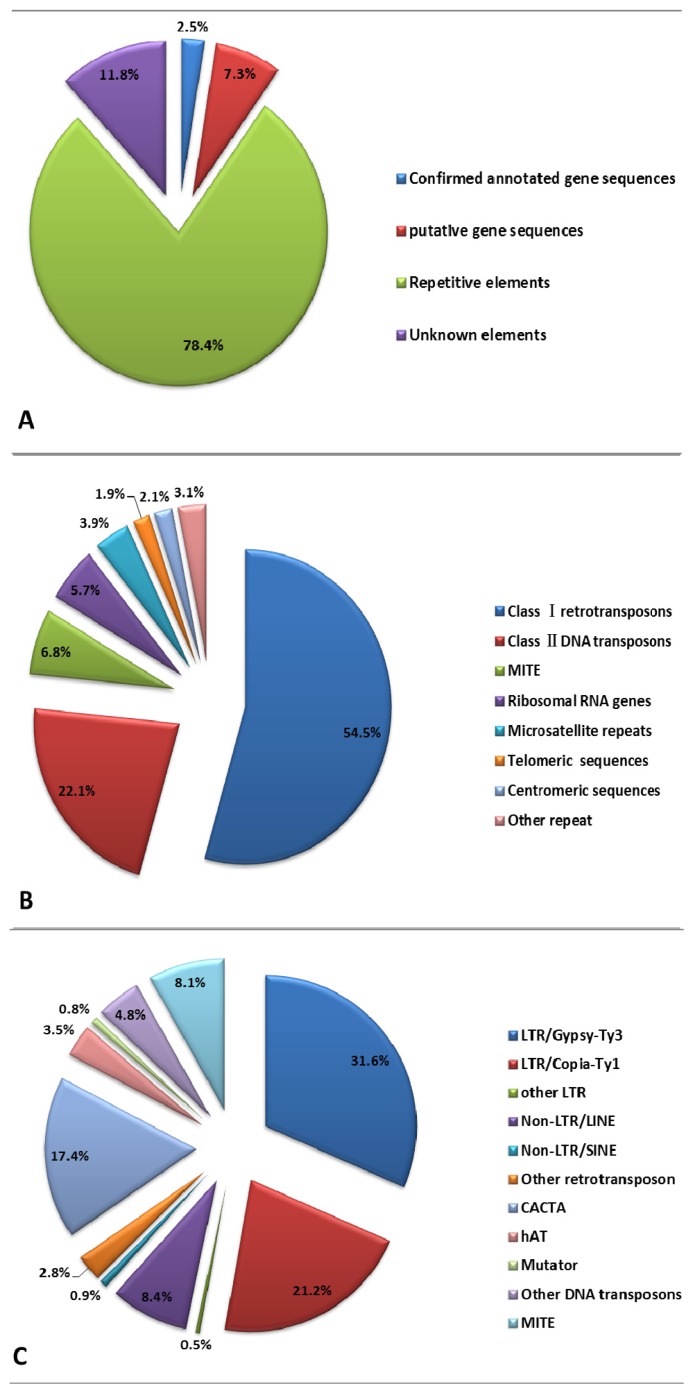
(**A**) Composition of the whole sampled ENS dataset; (**B**) Composition of total repetitive DNA elements; (**C**) Composition of total TE repeats.

Sequencing of the 672 randomly selected clones generated 1075 assemblies (126 contigs, 949 singlets) with an average length of 674.43 bp, a total length of 725,008 bp and a GC% content of up to 39.00% (Table 1, Figure 1). All sequences were deposited into GenBank (Accession numbers: JY266095-JY267380). Out of the 1075 assemblies, 1023 contained nuclear DNA sequences, while 50 assemblies comprised organellar (chloroplast and mitochondria) DNA (Table 1). After excluding organellar and bacterial DNA sequences, the dataset of 1023 assemblies comprising 690,804 bp of nuclear DNA sequence was obtained and designated as the ENS (*E. sagittatum* nuclear sequence) dataset. This represents 0.0154% of the entire nuclear genome based on the approximated 1C-value of 4496 Mbp. Within the ENS dataset, at least 795 assemblies contained repetitive DNA elements with a calculated total length of 541,625 bp, representing 78.41% of the ENS dataset. The total length of the “confirmed annotated” gene sequences was 17,360 bp (2.51% of the ENS dataset) while the total length of “putative” gene sequences was 50,466 bp (7.3%). No sequence homology was detected for 130 assemblies using the analyses herein, so these were referred to as “unknown” (Table 2, Figure 2A).

### 2.2. Repetitive DNA Elements of *E. sagittatum* Genome

In total 451,355 bp (65.34% of the ENS dataset) of sequence showed significant homology to the repeats in Repbase or the TE-related proteins in either the NR protein database or the Plant Repeat Database. These were categorized as “total TE repeats”. In addition to the total TE repeats, ribosomal RNA genes (31,002 bp, 4.49%), microsatellite repeats (21,065 bp, 3.05%), telomeric sequences (10,249 bp, 1.48%), and centromeric sequences (11,105 bp, 1.61%), were identified, giving a total of 541,625 bp from 795 assemblies identified as repetitive DNA elements, occupying 78.41% of the ENS dataset (Table 3, Figure 2A). By extrapolation, this suggests that the total repetitive DNA content in the *E. sagittatum* genome may be as high as 78.41%.

TE repeats (Class I retrotransposons, ClassII DNA transposons and MITEs) accounted for 83.33% of the entire repetitive DNA content of *E. sagittatum*, (Table 3, Figure 2B). Of these, retrotransposons (Class I) were the dominant class, accounting for 65.37% of the total TE repeats, which could be further subdivided into *Gypsy-Ty3* LTR retrotransposons (31.59%), *Copia-Ty1* (21.20%), other LTRs (0.48%), Non-LTR/LINE 8.41%, Non-LTR/SINE 0.85% and other retrotransposons 2.83%. DNA transposons (Class II) totaled 119,536 bp, accounting for 26.48% of the total TE repeats, with DNA/*CACTA*, DNA/*hAT*, and DNA/*Mutator* the most abundant (Table 4, Figure 2C).

### 2.3. Gene Content Analysis and Gene Number Estimation

Gene prediction using BlastX, FGENESH, and EST database support identified 23 “confirmed annotated” gene sequences (2.51% of the ENS dataset) and 75 “putative” gene sequences (7.31% of the ENS dataset) (Table 2). Most of the captured gene sequences were related to plant primary metabolism including an amino acid carrier, a secondary cell wall-related glycosyltransferase, beta-fructofuranosidase and 3-isopropylmalate dehydratase. The combined coding length of the 23 “confirmed annotated” gene sequences was 9275 bp, and was used to extrapolate the predicted protein-coding portion of the *E. sagittatum* nuclear genome. Based on the assumption that the average gene length is 2 kb, similar to Arabidopsis [38], the 9275 bp sequences implies a total of 60.23 Mbp protein-coding sequence within the entire nuclear genome, and a total gene number of 30,114 was predicted.

### 2.4. Chromosomal Number Analysis of *E. sagittatum*

**Figure 3:**
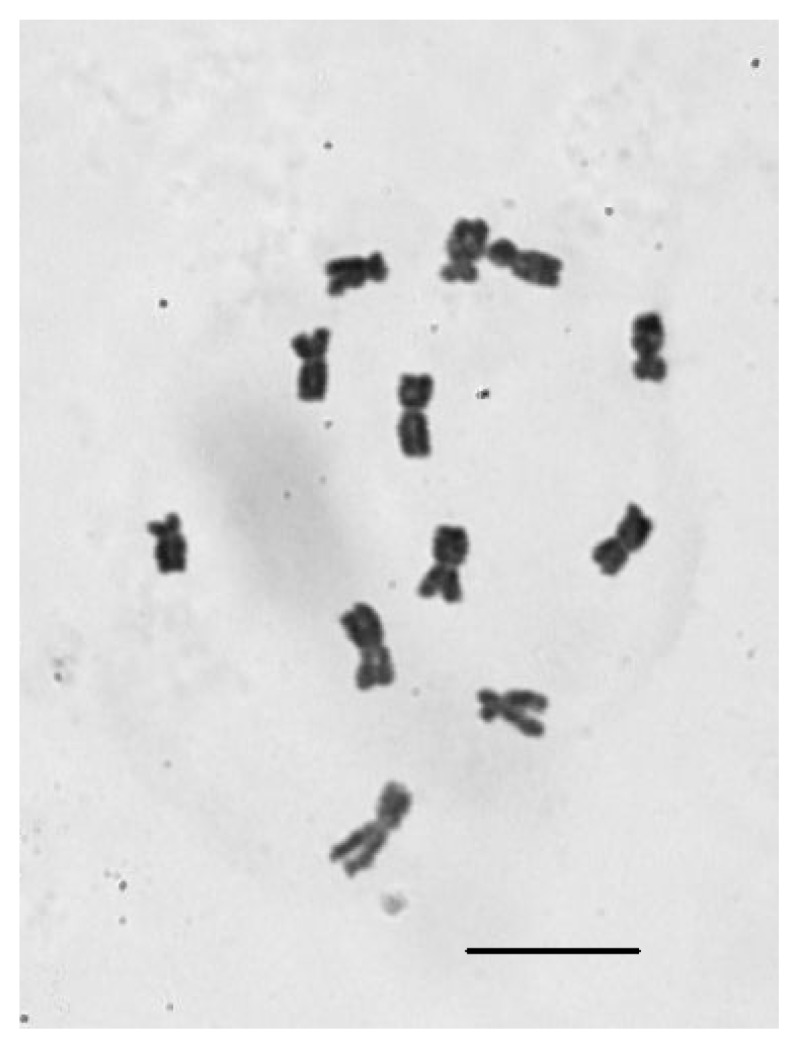
Root-tip mitotic metaphase chromosomes of *E. sagittatum* 2*n* = 2*x* = 12 are shown. Scale bar = 5 μm.

Chromosome counts of metaphase *E. sagittatum* cells revealed a chromosome number of 2*n* = 2*x* = 12 (Figure 3). This result is consistent with the observations of somatic chromosome number in additional *Epimedium* taxa by Zhang *et al.* (2008) [16].

### 2.5. Chromosomal Distribution of Gypsy-Ty3 Retrotransposons in *E. sagittatum*

**Figure 4:**
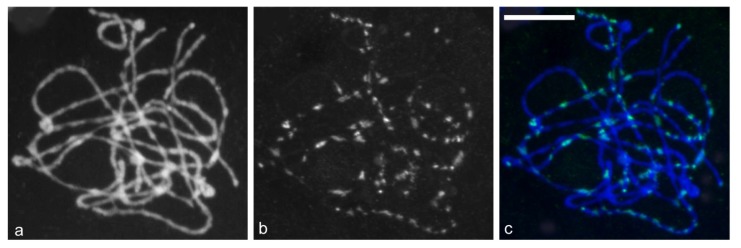
FISH analysis of *Gypsy-Ty3* retrotransposons distributed on pachytene chromosomes of *E. sagittatum*; (**a**) DAPI stained chromosomes; (**b**) Dispersed signal; (**c**) Merged images. Scale bar = 10 μm.

Given the prevalence of *Gypsy-Ty3*-type sequences in the genome sequence obtained for *E. sagittatum*, FISH to pachytene chromosomes of *E. sagittatum* was conducted using probes derived from *Gypsy-Ty3* reverse transcriptase (*rt*), to examine the distribution of major retrotransposable elements in the *E. sagittatum* nuclear genome. The FISH analysis revealed that *Gypsy-Ty3* retrotransposons are distributed unevenly on all chromosomes, and some strong signals were detected in the telomeric domains of several chromosomes (Figure 4). The extent of hybridisation suggested that *Gypsy-Ty3* retrotransoposons are prolific in the *E. sagittatum* genome.

## 3. Discussion

### 3.1. Characteristics of *E. sagittatum* Genome

*E. sagittatum* is a member of the Berberidaceae family of basal eudicot plant species recorded in the Chinese Pharmacopoeia as one of five *Epimedium* species with valuable medicinal properties. This study used the GenTrek approach, combined with chromosome and FISH analyses, to extrapolate and reveal novel insights into the genome sequence, structure and composition of *E. sagittatum*. This provides the first detailed genomic analysis of this evolutionarily and agriculturally important medicinal plant species.

In total, over 69,0804 bp of nuclear DNA, designated the ENS dataset, was sequenced from 1075 sequence contigs, representing 0.0154% of the *E. sagittatum* nuclear genome. A total of 23 sequences, representing 2.51% of the ENS dataset, were annotated as confirmed protein-coding sequences, while a significantly larger portion of the genome, comprising 78.41% of ENS dataset, was identified as repetitive DNA. Of this, the majority of repetitive DNA comprised transposable elements, and specifically Class I retrotransposons. This is consistent with previous investigations showing that retrotransposable elements dominate the repetitive DNA content of plant genomes, particularly LTR retrotransposons, whose amplification has been the major cause of genome expansion [39,40]. By randomly sequencing and annotating a small fraction of the whole genome, the GeneTrek approach has provided the first insight into the detailed genomic makeup of *Epimedium* spp, laying the foundations for the further elucidation of genome organization.

### 3.2. Genome Size Variation of *Epimedium* Species

The chromosomal number of all *Epimedium* species examined to date is 2*n* = 2*x* = 12 [16]. Consistent with this, our chromosome analysis revealed 12 chromosomes (2*n* = 2*x* = 12) for *E. sagittatum*. Despite this relatively small chromosome number, *Epimedium* species have intermediately-sized genomes compared to other angiosperms [41], with haploid genome sizes ranging from 4115.35 Mbp to 4876.4 Mbp [17]. According to the plant DNA C-values database [42], angiosperm genomes vary over 2,400-fold in size, from 63 Mbp/1C in *Genlisea aurea* [43] to 148,852 Mbp/1C in *Paris japonica* [44]. Within the Berberidaceae, the largest genome currently recorded is 14,351 Mbp, for *Podophyllum emodi* [45], while other Berberidaceae species vary from 489 Mbp in *Berberis koreana* [46] to 4005 Mbp in *Epimedium alpinum* [47]. Intriguingly, significant genome size variation in *E. sagittatum* was observed between different geographical populations [17]. This is consistent with the belief that intraspecific genome size variation is associated with adaption to different growing conditions or habitat [48].

### 3.3. Repetitive DNA and Retrotransposable Elements

As mentioned above, 78.41% of the randomly sampled nuclear genomic sequences for *E. sagittatum* were identified as repetitive DNA. Therefore, by extrapolation it is suggested that at least 78.41% of the whole nuclear genome of *E. sagittatum* is repetitive DNA. Given that *E. sagittatum* is a relatively under-studied plant species, it is likely that this is an underestimation due to insufficient annotations of potentially novel species-specific repeats. Previous research demonstrated that repetitive DNA elements are important components of eukaryotic genomes and play a significant role in genome size variation and genome evolution. For example, it is well established that the differences in genome size observed in the plant kingdom are accompanied by variations in the amount of repetitive DNA. In maize for example, TE families accounted for 70% of the genome size variation between the cultivated B73 inbred line and its related species *Zea luxurians* [49]. More specifically, this has been attributed to the amplification and deletion of LTR retrotransposons, suggesting that these elements are important players in the evolution of plant genome size and polyploidy [40,50–52]. In B73 maize, TEs constitute over 85% of the genome, with 75% of this attributed to LTR retrotransposons [53]. LTR retrotransposons also account for the majority of the nuclear genome in many other plant species [30,31,33], including species with relatively small genomes and proportionally less repetitive DNA [32,35,54]. Consistent with these findings, LTR retrotransposons comprised the majority of repetitive DNA in the sampled *E. sagittatum* genome. Of these, *Gypsy-Ty3* retrotransposons were the dominant type, being almost two times more abundant than *Copia-Ty1* type sequences (Table 4, Figure 2C). In cotton and rice, the ratio of *Gypsy-Ty3* to *Copia-Ty1* retrotransposons was reported to be around 2:1 [31,55], while it was found to be around 1:1 in Arabidopsis [38] and maize [28]. Therefore, it appears that the ratio of *Gypsy-Ty3* to *Copia-Ty1* retrotransposons is similar in *E. sagittatum* to cotton and rice.

In tomato BACs [56] and the genus *Helianthus* and other Asteraceae [57], *Gypsy-Ty3* has been shown to preferentially localize to centromeric or pericentromeric chromosome regions. However, *Copia-Ty1* retrotransposons were distributed unevenly and mostly at the precentromeric and terminal heterochromatin regions [17]. The different content and localization of *Gypsy-Ty3* and *Copia-Ty1* retrotransposons in plant genomes suggests that these two retrotransposons effect genome size variation and genome evolution independently and may have distinct roles in the evolutionary history of genome expansion. Analysis of the diversity of *copia*-RT fragments in *Epimedium* revealed relatively low *copia*-RT sequence heterogeneity, suggesting that *Copia-Ty1* retrotransposons experienced bursts of activation followed by deactivation during *Epimedium* genome evolution, leading to rapid increases in copy number and subsequently rapid increases in genome size [17]. This study has contributed our understanding of the TE composition of the *Epimedium* genome, and the potential roles of various TEs in genome size evolution, however more in-depth efforts are needed to explore retrotransposon behavior in detail and the impact of these TEs on plant genome size, composition and evolution.

### 3.4. Gene Content Analysis

Extrapolation of the number and cumulative length of “confirmed annotated” genic sequences from the sampled genomic data predicts the total gene number of *E. sagittatum* to be 30,114. Comparisons to species with known genome sizes and gene numbers suggest this is a reasonable estimate for *Epimedium* spp. For example, 27,411 genes are estimated in Arabidopsis (TAIR, version 10, Department of Plant Biology, Carnegie Institution: Stanford, CA, USA, available on http://www.arabidopsis.org/index.jsp), 39,045 genes in rice [58], 24,746 genes in papaya [59], 45,555 genes in black cottonwood [22], 30,434 genes in grapevine [60], 32,000 genes in maize[53], 34,809 genes in woodland strawberry[61], 40,976 genes in diploid cotton [62] and 34,727 genes in tomato [24]. Nonetheless, gene prediction is challenging in plants and other complex eukaryotic genomes and many transposable elements in plant BACs and genomes have been annotated as hypothetical genes, leading to a consistent over-prediction of gene number [63]. As such relatively stringent criteria were used to predict the total gene number in *E. sagittatum*.

The consistency in repetitive element and gene content estimates between *Epimedium* herein and published plant genomes supports suggestions that the GeneTrek approach is a cost-effective and efficient way to gain a global insight into relatively large plant genomes. Previous GeneTrek analysis of the maize genome predicted 37,000 genes [27] and at least 66% repetitive DNA [29]. Accordingly, the draft sequence of the whole maize genome predicted over 32,000 genes, and revealed that nearly 85% of the genome is composed of transposable elements [53]. In future, the sampling of more *E. sagittatum* genomic sequences may further narrow-down the range of estimated gene number for *E. sagittatum* [35]. Furthermore, based on the sequence data for the *E. sagittatum* genome provided herein, future work could focus on the mining of genes involved in secondary metabolite biosynthesis and examining their regulatory factors.

## 4. Experimental Section

### 4.1. Plant Material

*E. sagittatum* was grown in Spring (March–June) in the field at the Wuhan Botanical Garden, CAS, China. Several fully-expanded leaves were harvested for DNA preparation from 25 days old plants. Root tips of the same plants were collected for chromosome analysis and young panicles were harvested for FISH analysis.

### 4.2. DNA Preparation and Construction of the Shotgun Insertion Clones

DNA was isolated following Rabinowicz’s protocol [64] and sonicated at 20% energy for 6 s and at 100% energy for 60 s in succession (Sonopuls GM 200, Bandelin, Berlin, Germany). Sonicated DNA was incubated with T4 DNA polymerase (Roche, Basel, Switzerland) and dNTPs (Takara, Dalian, China) at 16 °C for 18 h to create blunt-ended fragments. DNA fragments were size separated on a 1% TAE agarose gel and fragments ranging from 0.3 kb to 2 kb were purified (Qiagen) and ligated at 16 °C for 18 h into dephosphorylated, *Sma*I-digested pBluescriptKS (+) vector (National Center for Gene Research, CAS, Shanghai, China) using the T4 DNA ligation kit (Roche, Basel, Switzerland). Recombinant plasmids were electroporated into *Escherichia coli* DH10B competent cells (National Center for Gene Research, CAS, Shanghai, China). Positive transformants were selected on LB-agar plates containing ampicillin (100 μg mL^−1^), IPTG (60 μg mL^−1^) and X-gal (24 μg mL^−1^), then incubated in glycerol storage medium in 384-well plates for storage at −80 °C. In total, 1536 plasmid clones were constructed.

### 4.3. Sequencing and Sequence Assembly

Plasmid DNA isolation was performed as per Sambrook and Russell (2001) [65]. In total, 672 randomly selected clones were sequenced bidirectionally with SP6 (5′-ATT TAG GTG ACA CTA TAG-3′) and T7 (5′-TAA TAC GAC TCA CTA TAG GG-3′) primers using the BigDye^®^ Terminator v3.1 Cycle Sequencing Kit (Applied BioSystems, Foster City, CA, USA) and an ABI 3730 capillary sequencer (National Center for Gene Research, CAS, Shanghai, China). Basecalling was performed with PHRED [66], sequence trimming and vector sequence removal were performed with TRIMMING [67] and SeqClean (http://compbio.dfci.harvard.edu/tgi/software/) using default settings, respectively. Sequence reads were assembled using CAP3 (Version Date: 12/21/07) [68]. The computational biology and functional genomics laborory

### 4.4. Sequence Annotation

#### 4.4.1. Identification of Repetitive DNA Elements

Given the lack of a reference repeat database for *Epimedium*, repetitive DNA elements were identified using Protein-Based Repeat-Masking in RepeatMasker [69] and also using CENSOR [70]. In addition, sequences of TE elements or TE related proteins which may not be detected by RepeatMasker [69] and CENSOR [70] were identified by a default Blastn search against the Plant Repeat Database at Michigan State University (http://plantrepeats.plantbiology.msu.edu/index.html) [71], and default BlastX (Expect value ≤10^−10^) searches against the National Center for Biotechnology Information non-redundant protein database [72]. The fraction of the genome that each class of repetitive DNA element represented was calculated as the ratio of the total length of repeat sequence to the total length of the *E. sagittatum* nuclear sequence (ENS) assembly, excluding the organellic sequences.

#### 4.4.2. Analysis of Gene Content

Gene content analysis was performed in three stages. First, a default BlastX (Expect value ≤10^−10^) search was performed against the NCBI (http://www.ncbi.nlm.nih.gov) non-redundant protein database (NR) [72]. Secondly, *ab initio* gene prediction was performed on the ENS dataset using the FGENESH feature (Dicot plants-Arabidopsis) of the MolQuest software package (softberry) [73]. Thirdly, a local BLAST to an in-house EST database of *E. sagittatum* was executed (Expect value ≤10^−10^). All sequence results were manually examined and evaluated and any showing homology to TE elements, TE related proteins or organelle proteins in the NR protein database were omitted. Among the remaining sequences, those both with significant homology in the NR protein database (Expect value ≤10^−10^, identity ≥60%, alignment length of amino acids ≥67), and supported by either the in-house EST search or FGENESH prediction, were designated as “confirmed annotated” gene sequences. Those sequences with significant homology in the NR protein database but without the EST or FGENESH support, and *visa versa*, were designated as “putative” gene sequence.

### 4.5. Chromosomal Analysis

*E. sagittatum* root tips were pretreated in a saturated solution of paradichlorobenzene (PDB) for 4 h, then fixed in Carnoy’s liquid fixative (ethanol:glacial acetic acid = 3:1) for 30 min at 4 °C before being macerated in 1*N*-hydrochloric acid for 6 min at 60 °C, stained with Carbol Fuchsin and then squashed for cytological observation.

### 4.6. FISH Analysis

Young panicles were fixed in Carnoy’s liquid fixative (ethanol:glacial acetic acid = 3:1) for 30 min at 4 °C. Microsporocytes at the pachytene stage were macerated in a 1:1 mixture of pectinase:macerozyme for 30 min and then squashed in 45% glacial acetic acid. The slides were dipped immediately into liquid nitrogen and stored at −70 °C. The probes originated from the reverse transcriptase (*rt*) sequence of *Gypsy-Ty3*, and were amplified from *E. sagittatum* DNA using degenerate primers (forward primer: 5′-TAYCCNHTNCCNCGNATHGA-3′; reverse primer: 5′-ARCATRTCRTCNACRTA-3′). Probes from pooled *E. sagittatum Gypsy-Ty3 rt* PCR fragments were labeled by the DIG-Nick Translation Mix Kit (Roche). FISH was carried out as per Jiang *et al.* (1995) [74]. Sheep Anti-Digoxigenin (Roche) was used to detect the Digoxigenin-labeled probes, and amplified with FITC-conjugated Anti-sheep IgG (Vector Laboratories, Burlingame, CA, USA). Chromosomes were counterstained in 30 μL 4′,6-diamidino-phenylindole (2 μg mL^−1^; DAPI, Sigma, St. Louis, MO, USA) in antifade solution (Vector). Images were captured by an Olympus BX61 fluorescence microscope with a Photometrics SenSys CCD (charge coupled device) 1400E. Greyscale images were merged by Metamorph software (Version 7.0, Molecular Devices: Sunnyvale, CA, USA).

## 5. Conclusions

*E. sagittatum* is a Chinese traditional medicinal plant with great potential in the development of modern and natural pharmaceuticals. Thus further research into this species is warranted in order to elucidate the genomic and biochemical basis of its medicinal properties and exploit these properly. Here we provided the first insights into the composition, structure and evolution of the *E. sagittatum* genome. Our results indicate that at least 78.41% of the whole genome consists of repetitive DNA elements, with LTR retrotransposons dominating, and thus may play a significant role in *Epimedium* genome evolution. Furthermore, we provide a reasonable gene number estimation of 30,114 genes. This study will pave the way for further functional genomic analysis of this valuable medicinal plant with regards to genome organization, species evolution and the function of genes involved in its complex metabolism.

## Figures and Tables

**Table 1:** Summary of the assemblies.

Total number of assemblies	1075
Contigs	126
Singlets	949
Total sequence length	725,008 bp
Average insert length	674.43 bp
GC content	39.00%
Nuclear DNA insert	1023
Organellic DNA insert (cp, mt) a	50
Bacterial DNA insert	2

a cp, chloroplast; mt, mitochondrion.

**Table 2:** Details of the sequence composition of nuclear DNA sequences.

Nuclear DNA insert	Number of assemblies	Calculated sequence length (bp)
Confirmed annotated gene sequences	23	17,360
Putative gene sequences	75	50,466
Repetitive DNA elements	795	541,625
Unknown elements	130	81,353
Total ENS dataset	1023	690,804

a ENS, *E. sagittatum* Nuclear Sequences.

**Table 3:** Summary of repetitive DNA elements.

	Number of assemblies	Calculated length of repetitive DNA (bp)	Percentage of total repetitive DNA (%)
Total TE a repeats	656	451,355	83.33
Classi retrotransposons	431	295,036	54.47
Class ii DNA transposons	171	119,536	22.07
MITE b	54	36,783	6.79
Ribosomal RNA genes	47	31,002	5.72
Microsatellite repeats	34	21,065	3.89
Telomeric sequences	16	10,249	1.89
Centromeric sequences	17	11,105	2.05
Other repeat	25	16,849	3.11
Total repetitive DNA elements	795	541,625	100

a TE, Transposable Element;

b MITE, Miniature Inverted-repeat Transposable Element.

**Table 4:** Detailed classification and composition of TE repeats.

	Number of assemblies	Calculated length of TE repeats (bp)	Percentage of TE repeats (%)
Classiretrotransposons	431	295,036	65.37
LTR a/*Gypsy-Ty3*	211	142,593	31.59
*Copia-Ty1*	136	95,698	21.20
Other LTR	3	2,176	0.48
Non-LTR LINE b/*RTE*	26	17,700	3.92
LINE/*L1*	16	11,985	2.66
Other LINE	13	8261	1.83
SINE c	6	3840	0.85
Other retrotransposon	20	12,783	2.83
Classii DNA transposons	171	119,536	26.48
*CACTA*	110	78,494	17.39
*hAT*	21	15,805	3.50
*Mutator*	6	3760	0.83
Other DNA transposons	34	21,477	4.76
MITE	54	36,783	8.15
Total TE repeats	656	451,355	100

a LTR, Long Terminal Repeat;

b LINE, Long Interspersed Nuclear Element;

c SINE, Short Interspersed Nuclear Element.

## Abbreviations

ENS: *Epimedium sagittatum* Nuclear Sequences
TE: Transposable Element
LTR: Long Terminal Repeat
LINE: Long Interspersed Nuclear Element
SINE: Short Interspersed Nuclear Element
MITE: Miniature Inverted-repeat Transposable Element.
